# Research on magnetic bead motion characteristics based on magnetic beads preset technology

**DOI:** 10.1038/s41598-021-99331-8

**Published:** 2021-10-07

**Authors:** Zhao Li, Xiangyang Zu, Zhe Du, Zhigang Hu

**Affiliations:** 1 Department of Packaging Engineering, Henan University of Science and Technology, Luoyang, Henan China; 2 School of Medical Technology and Engineering, Henan University of Science and Technology, Luoyang, Henan China

**Keywords:** Biotechnology, Biomedical engineering

## Abstract

In order to improve the detection efficiency and accuracy of microfluidic chip, a magnetic beads preset technology were designed by using double permanent magnets as external magnetic field and the motion characteristics of preset magnetic beads were studied. The control principle of magnetic beads preset technology was introduced in detail, and the control structure was designed. The coupled field characteristics for magnetic beads in microchannels were analyzed, and the motion models of magnetic beads were established based on the magnetic beads preset technology, including capture motion and mixing motion. The relationship between the magnetic field force and the flow velocity for capturing magnetic bead, and the mixing time under the influence of flow field and magnetic field were derived. The magnetic beads preset technology effect was verified by experiments and numerical simulations were developed to analyze the influence of aspect ratio of permanent magnet on magnetic field. The study showed that the accuracy and efficiency of the magnetic bead control in the microchannel could be better realized by the magnetic beads preset technology. The derivation of the magnetic bead motion model can understand the motion characteristics of the magnetic bead more clearly, facilitate accurate control of the magnetic bead, and improve the success rate of the microfluidic detection.

## Introduction

Magnetic bead is a kind of magnetic material with particle size between 1 and 100 nm, which not only has unique surface effect, volume effect, quantum size effect, functional groups like other general nano material, but also exhibits superparamagnetism, magnetic responsiveness, high coercivity^1^, and can be controlled by external magnetic field. At present, magnetic bead is attracting more and more attention^2–5^, and is widely used in biomedical science^6–11^. For example, the magnetic beads with special surface treatment can be used to form a new complex by nonspecific or specific combination with the corresponding target materials, and can be separated under the control magnetic field. Then the separated magnetic beads are eluted by the eluent to complete DNA extraction^12,13^. The key of magnetic bead control is to set a reasonable magnetic field to achieve the effect that magnetic beads can be effectively captured and fully mixed with the reaction solution under the flow field influence.

Numerous of methods have been reported in the literature on controlling magnetic beads to improve magnetic beads capture efficiency and mixing efficiency. Zhou et al.^14^ integrated an iron–PDMS microstructure next to a microfluidic channel and placed between two external permanent magnets by injecting a mixture of iron powder and PDMS into a prefabricated channel. The magnetized iron–PDMS microstructure generates localized and strong forces on the magnetic particles to enhanced separation of the particles. Zhu et al.^15^ built a specific magnetic field in a desired chamber bonded with PDMS and PMMA sheets by embedding soft magnets of iron powders in the chip, and magnetized by an external magnetic field to form the nucleic acid capture platform based on magnetic beads. By improving the liquid flow rate and magnetic field distribution, the nucleic acid extraction efficiency was increased by about 80%, and the serpentine flow channel was used to increase the mixing efficiency of magnetic beads and reaction solution. Serra et al.^16^ realized the construction of high-throughput sequencing library based on microfluidic chip and the capture of nucleic acids of different sizes by integrating soft magnetic structure and pressure control valve. This method realized higher nucleic acid extraction efficiency (> 96.5%) than the traditional magnetic bead method, with the application advantages of high-throughput, automation and compatibility with downstream sequencing. Munaz et al.^17^ developed a simple microfluidic platform for separating diamagnetic particles with different sizes by using parallel ferromagnetic fluid, so that ferromagnetic fluid with predetermined concentration of magnetic nanoparticles can promote negative magnetophoresis. The result shows that the maximum separation efficiency of particles for 3.2 and 4.8 μm was 78% and 75% respectively. Zhang et al.^18,19^ both designed a special 3D structure in the microfluidic chip to improve the collision probability between magnetic beads and reaction liquid, so as to improve the mixing efficiency.

Current research focuses on the design of control magnetic field, especially how to increase the magnetic field gradient to enhance the magnetic field force and improve the capture efficiency, and how to increase the length and complex of flow channel to improve the mixing efficiency. And the magnetic beads are mostly introduced from the outside of microfluidic chip, so the magnetic beads control system is usually complex, which is not conducive for the popularization of microfluidic detection technology. In addition, the motion characteristics of magnetic beads is the basis of the control magnetic field design, which is an important research direction of microfluidic detection.

This paper presents a magnetic beads preset technology, and analyzes the feasibility of magnetic beads preset technology and the motion characteristics of preset magnetic beads, in order to improve the efficiency of microfluidic detection and simplify the microfluidic detection equipment.

## Analysis of magnetic bead control

### Magnetic bead control motion

**Figure 1:**
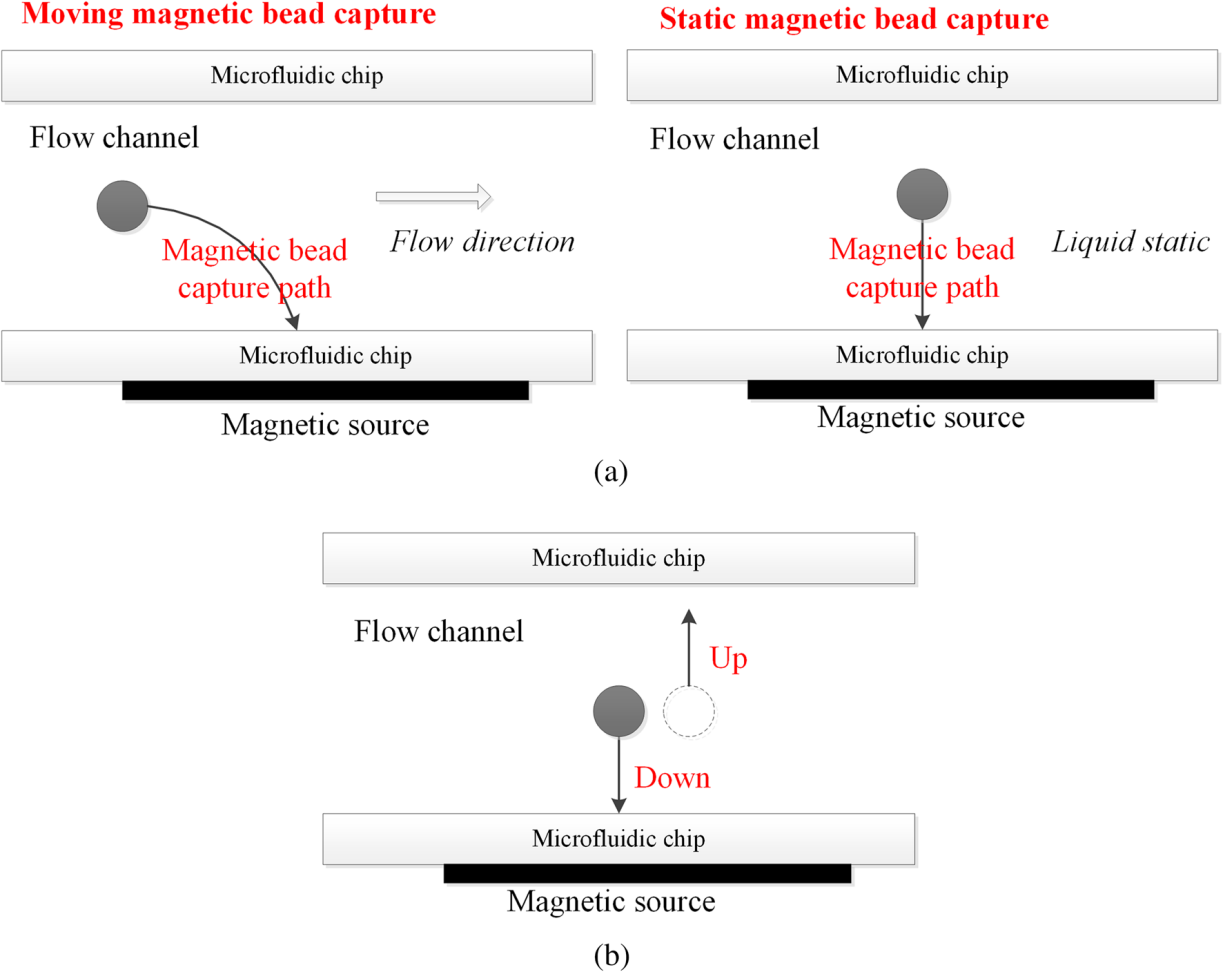
Magnetic bead control motion. (**a**) Magnetic bead capture. (**b**) Magnetic bead mixing with the solution.

The functions of magnetic beads are different in microfluidic detection, but what they all have in common is to realize the accurate control of magnetic beads in micro scale and enhance mass transfer efficiency by use of magnetic field. Therefore, the dynamic characteristics and transmission pattern of magnetic beads in the micro channel under the effect of magnetic field are important research on the control of magnetic beads^20–22^. This paper focuses on the control motions about magnetic beads capture and magnetic beads mixing with the solution, which involves two problems: the capture efficiency and mixing efficiency, as shown in Fig. 1.

#### Capture efficiency

Magnetic beads are generally exogenous for microfluidic chips, thus it is necessary to capture the magnetic beads to the designated position in chip channel and wait for the subsequent reactions, as shown in Fig. 1a, including moving magnetic capture and static magnetic capture. The magnetic beads capture efficiency mainly includes the number of magnetic beads captured and the time required to complete the capture. The effective number of magnetic beads captured can ensure the success rate of detection, and the reasonable capture time can improve the detection efficiency. Magnetic beads capture efficiency is an important parameter in micro scale biological detection, which is mainly affected by gradient of magnetic force^23^ and fluid viscous resistance^24^. In our study, the magnetic beads were used to be endogenous of microfluidic chip, which can fully ensure magnetic beads capture efficiency.

#### Mixing efficiency

The magnetic beads mixing motion refers to the relative motion between magnetic beads and reaction solution, as shown in in Fig. 1b. Because the Reynolds number of fluid in microchannel is very small (generally less than 10), the conventional turbulent mixing will not occur. If the completion of fluid mixing only depends on diffusion without external disturbance, the mixing time will be too long and the mixing efficiency will be very low. Too long mixing time will result in low efficiency of the detection system, and insufficient mixing will affect the accuracy of the detection system. According to whether there is external power, the mixing process of magnetic beads and solution can be divided into passive mixing and active mixing. Compared with passive mixing, the mixing effect of active mixing is more satisfactory and easy to control, so the active mixing is used as the control method of magnetic beads mixing motion in our study.

### Selection of control magnetic field

According to the differences of magnetic field sources, the research about magnetic bead control technology is mainly divided into two categories: micro-electro-mechanical system (MEMS) and external permanent magnet system. Generally speaking, although the MEMS technology has higher integration, the following problems^25,26^ exist: (1) the integrated coil will generate heat, and a new heat source will be introduced on the chip, which will be difficult to adapt to the temperature sensitive sample; (2) the magnetic induction generated by MEMS is not high, so the separation efficiency is low; (3) the processing of MEMS is complicated and difficult; (4) the chip used in MEMS is usually opaque, which will be unfavorable for optical detection. However, the external permanent magnet system is relatively simple, which will not affect microchannel design and will be easy to achieve magnetic field control. In our study, a kind of external permanent magnet system was designed and the motion characteristics of magnetic beads based on the system were analyzed.

## Magnetic beads preset technology

Magnetic beads preset technology, mainly including the method of magnetic beads preset and the control structure of preset magnetic beads, is to pre place the magnetic beads into the microfluidic chip, and in this way, magnetic beads can directly participate in the reaction when the reaction occurs.

### Magnetic beads preset microfluidic detection process

Microfluidics is a technology using microchannels with sizes of 10–100 μm to handle or manipulate microfluidics^27,28^. Microfluidics detection is a micro total analysis system completing a series of biochemical experiments on a microchip, such as biological samples preparation, DNA extraction, PCR reaction, on-chip detection and so on^29–31^. Compared with traditional detection methods, microfluidic detection is obviously advantageous. Microfluidic can handle a small quantity of samples to speed up the synthesis and analysis and reduce the consumption of reagents. The high surface area mass ratio and heat transfer coefficient of microfluidic can significantly shorten the time required for reaction and analysis to improve detection efficiency. The laminar flow characteristics in microfluidics can accurately control and optimize the fluid motion. Microfluidics devices which are compact, miniaturized, highly automated, and allow parallel analysis, can achieve high-throughput, large-scale and rapid detection.

**Figure 2:**
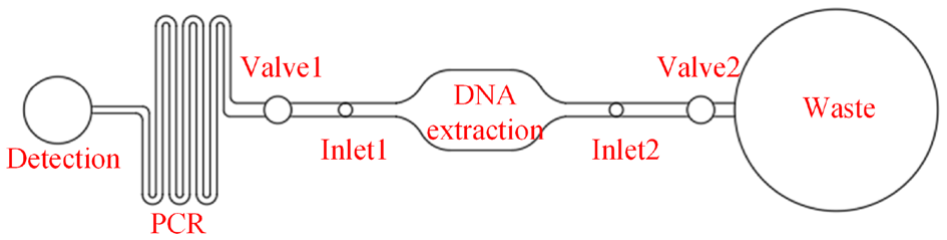
Structure of microfluidic chip with preset magnetic beads.

The platform of microfluidic detection is microfluidic chip^32–34^. The structure of microfluidic chip designed with preset magnetic beads is shown in Fig. 2. The detection process is as follows: (1) close valve 1 and open valve 2, the distilled water was injected from inlet 1, then the preset magnetic beads in the DNA extraction area began to dissolve and release. After magnetic beads completely releasing and capturing, the sterile air was introduced into microchannel from inlet 1 for purging; (2) close valve 2 and open valve 1, sample was injected from inlet 2 and fully mixed with magnetic beads by the control of magnetic field; (3) close valve 1 and open valve 2, sterile air was introduced into microchannel again from inlet 1 for purging; (4) close valve 2 and open valve 1, eluent was injected from inlet 2 and fully mixed with magnetic beads by the control of magnetic field; (5) close valve 2 and open valve 1, the pushing liquid was injected from inlet 2 to push the eluent to the PCR reaction area; (6) after the eluent completing the PCR reaction, the eluent was pushed to the detection pool by pushing liquid for fluorescence detection; (7) display the detection results, and complete the microfluidic detection.

### Magnetic beads preset method

**Figure 3:**
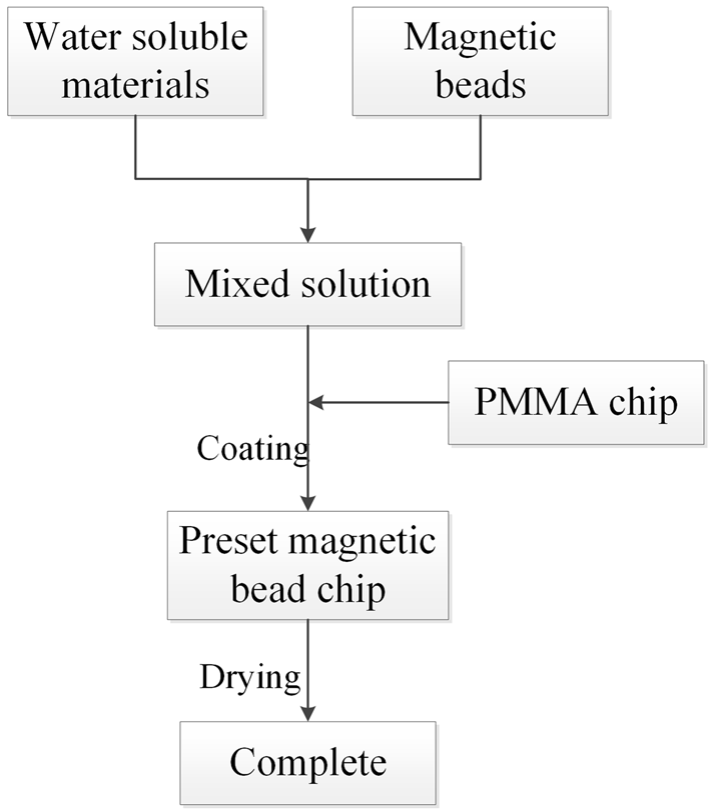
Magnetic beads preset process.

The process of magnetic beads preset is shown in Fig. 3, and the detailed implementation steps are as follows.

1. Preset coating mixing. Put coating solution (such as PVA) and magnetic beads into vortex oscillator to fully mix to ensure mixing solution uniform density.
2. Preset coating spraying. The quantitative mixing solution was evenly coated on the bottom of magnetic beads preset groove on the chip, and the thickness of the magnetic beads preset coating can be adjusted as needed, but it should not exceed 1/3 of the magnetic bead preset groove depth.
3. Preset coating drying. Place the chip with preset coating into the blast drying oven at the temperature of 50–70 ℃ for 20–30 min. After the water in PVA solution was volatilized, the magnetic beads preset coating will be formed, which can wrap magnetic beads and effectively adhere to the bottom of magnetic bead preset groove.
4. Microfluidic chip assembly. Make the film bonding on the chip to complete preset magnetic beads encapsulation, and paste the permanent magnet on the back of the chip to complete the assembly of magnetic beads preset microfluidic chip.

### Preset magnetic beads control structure

The functions of preset magnetic beads control structure mainly include the capture of preset magnetic beads during the dissolution of magnetic beads coating and the full mixing between preset magnetic beads and reaction solution.

#### Preset magnetic beads capture

**Figure 4:**
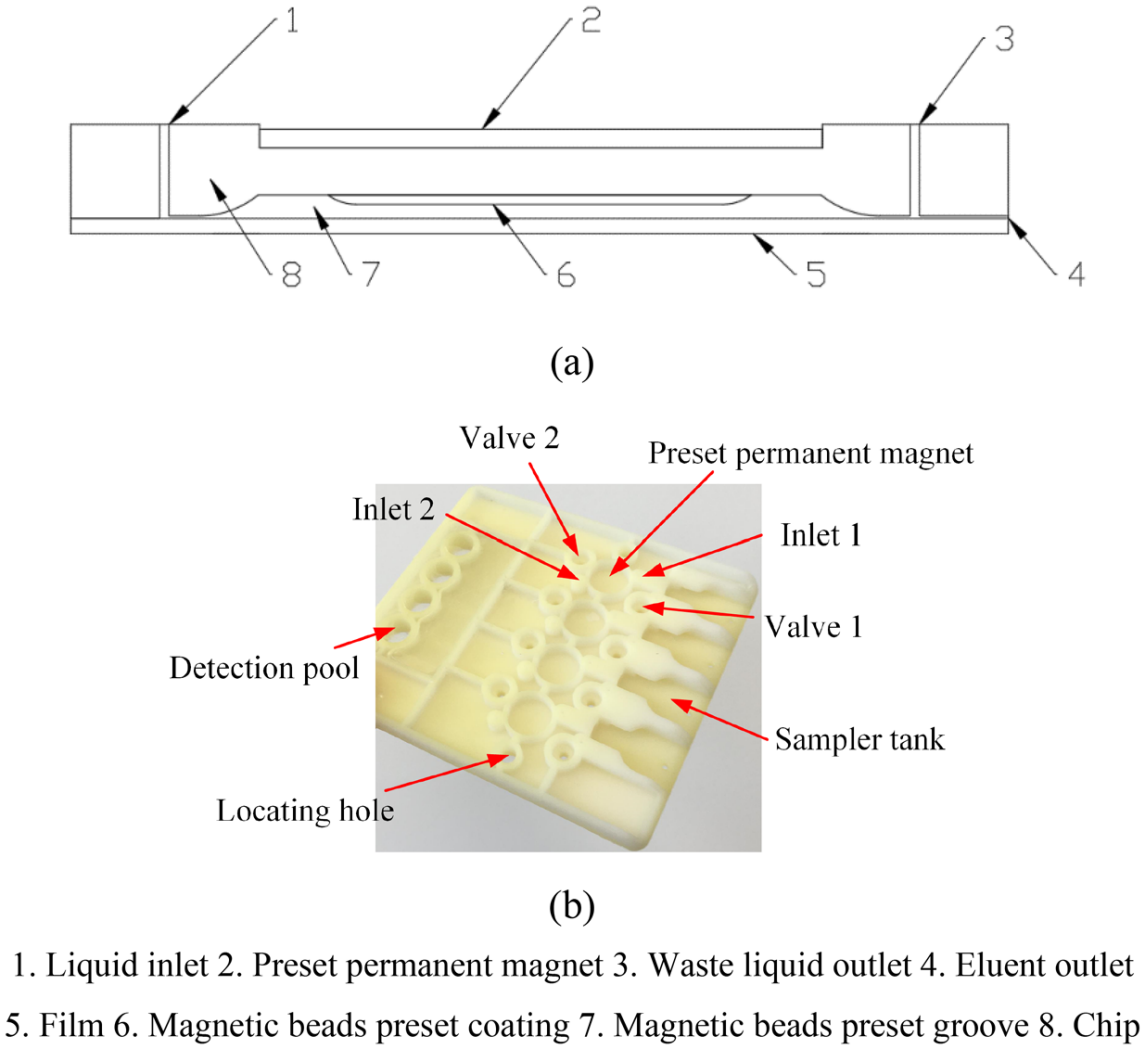
Magnetic beads preset microfluidic chip. (**a**) Structural schematic diagram. (**b**) Chip structure.

Figure 4a shows the structure of microfluidic chip with preset magnetic beads. When the detection began, distilled water was injected into and filled the magnetic beads preset groove 7 from liquid inlet 1 with eluent outlet 4 closed by valve (not shown in the figure) and the waste liquid outlet 3 opened. So the magnetic beads preset coating 6 would be placed in distilled water and after a while, the water-soluble material in the magnetic beads preset coating was dissolved, then the magnetic beads wrapped were released. Due to the magnetic force of preset permanent magnet 2, the magnetic beads were captured at the bottom of magnetic beads preset groove. The chip structure is shown in Fig. 4b.

#### Preset magnetic beads mixing

**Figure 5:**
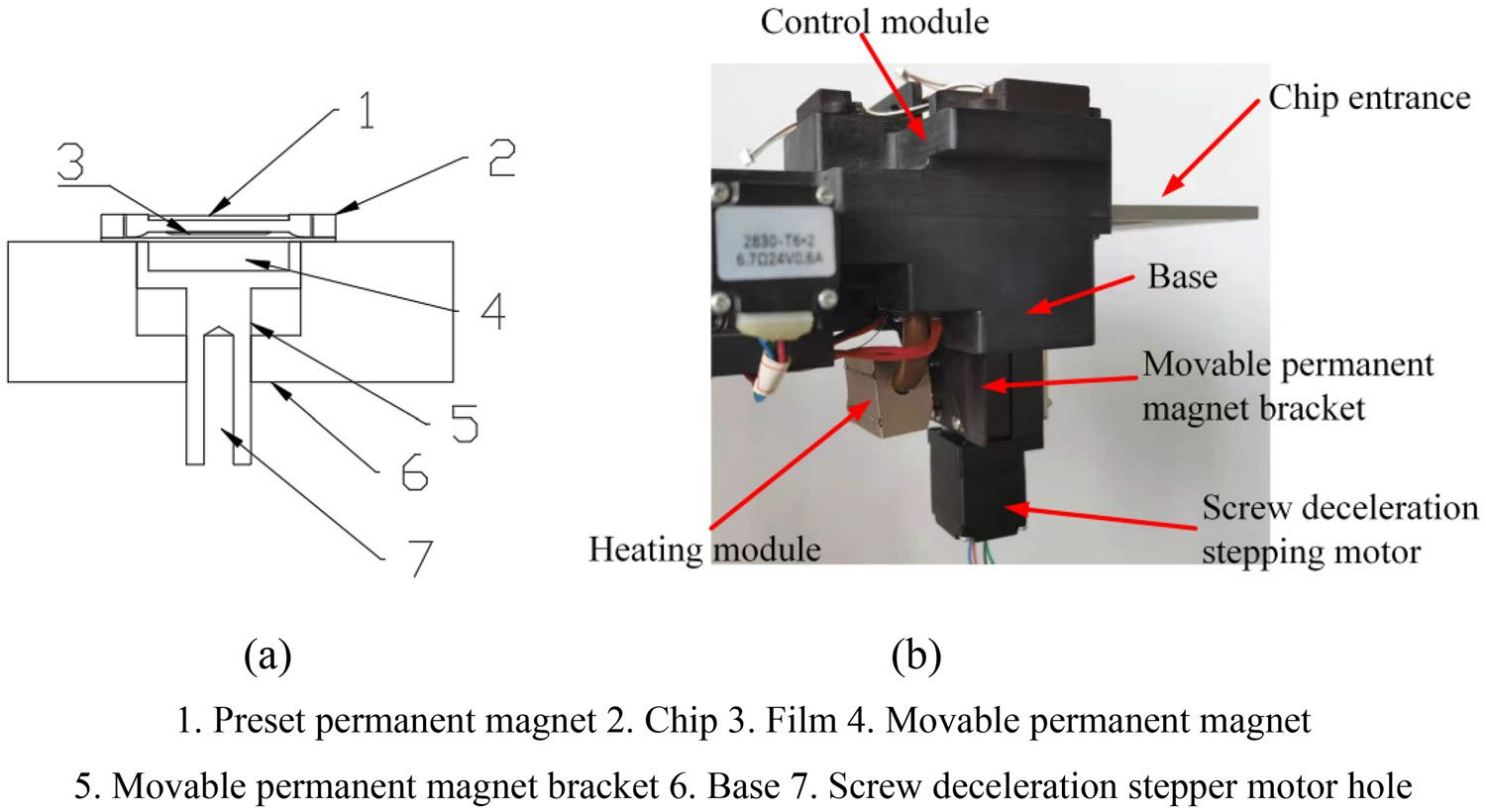
Magnetic beads mixing motion control structure. (**a**) Structural schematic diagram. (**b**) Motion platform.

Full mixing of magnet beads and adsorbent (sample solution) or eluent is the premise to ensure the effect of adsorption or elution. Magnetic beads preset technology adopted double-sided permanent magnet system, and the magnetic beads mixing motion was realized by the difference of magnetic field intensity caused by the interaction between preset permanent magnet (fixed weaker permanent magnet) and the movable permanent magnet (stronger permanent magnet). As shown in Fig. 5a, magnetic beads preset microfluidic chip was fixed on the base 6 with a motion guide groove for the movable permanent magnet bracket 5 to ensure the up and down motion of movable permanent magnet bracket. The up and down motion of movable permanent magnet 4 was achieved by screw deceleration stepping motor (not shown in the figure), which cooperated with the thread hole at the lower end of movable permanent magnet bracket 5.

After the magnetic beads preset coating was dissolved, the magnetic beads were forced on the bottom of magnetic beads preset groove by preset permanent magnet 1, and the initial position of movable permanent magnet 4 was located at the bottom of base guide groove, so the influence of movable permanent magnet on the magnetic beads could be ignored. After the adsorbent or eluent was filled, the movable permanent magnet bracket 5 and the movable permanent magnet 4 were raised. Because the magnetic field strength of movable permanent magnet was much stronger than that of the preset permanent magnet, the adsorption force of movable permanent magnet for the magnetic beads gradually increased with the rise of movable permanent magnet, and the magnetic beads began to leave the bottom of the magnetic beads preset groove and moved towards the film direction, which will realize the downward mixing of the magnetic beads with the adsorbent or eluent. When the movable permanent magnet moved downward, the adsorption force for magnetic beads gradually weakened, and the adsorption force of preset permanent magnet for magnetic beads became the main adsorption force again. The magnetic beads moved to the bottom of magnetic beads preset groove, which realized the upward mixing between the magnetic beads and the adsorbent or eluent. The control of magnetic beads mixing motion could be adjusted by changing the motion speed for adjusting mixing speed. The motion track of magnetic beads can be controlled by changing the size of movable permanent magnet. The suspension distribution of magnetic beads can be realized by changing the relative position of movable permanent magnet and preset permanent magnet. Magnetic beads mixing motion control platform is shown in Fig. 5b.

### Advantages of magnetic beads preset technology

#### Achieving the accurate control of magnetic beads using amount

The coupling effect between flow field and magnetic field should be considered for magnetic beads capture in microfluidic chip, and the motion of magnetic beads capture is complex, and difficult to accurately control the amount of magnetic beads capture^35^. When the capture amount is insufficient, the detection requirements are hard to satisfy. When the capture amount is too large, the detection cost will increase, which will affect the promotion of microfluidic detection technology. The magnetic beads preset technology can accurately quantify the magnetic beads in the chip to effectively ensure the optimal amount of magnetic beads using for microfluidic detection.

#### Simplify microfluidic devices

Microfluidic detection involves the supply of different reaction liquid, the design of different mechanical control devices (such as pump, valve), and design of different field control systems (such as optical, thermal, magnetic field system). However, the size of microfluidic chip is very small, which leads to the higher integration of microfluidic detection device, so the operation structure design for microfluidic chip is more complex due to the limitation of space. And if a certain operation structure for microfluidic chip can be reduced, it will greatly simplify the microfluidic devices.

With magnetic beads preset technology, the magnetic beads are pre-placed in the chip, reducing the magnetic beads feedings mechanism and the complexity of microfluidic device.

## Analysis of magnetic beads preset technology effect

### Magnetic beads preset coating

**Figure 6:**
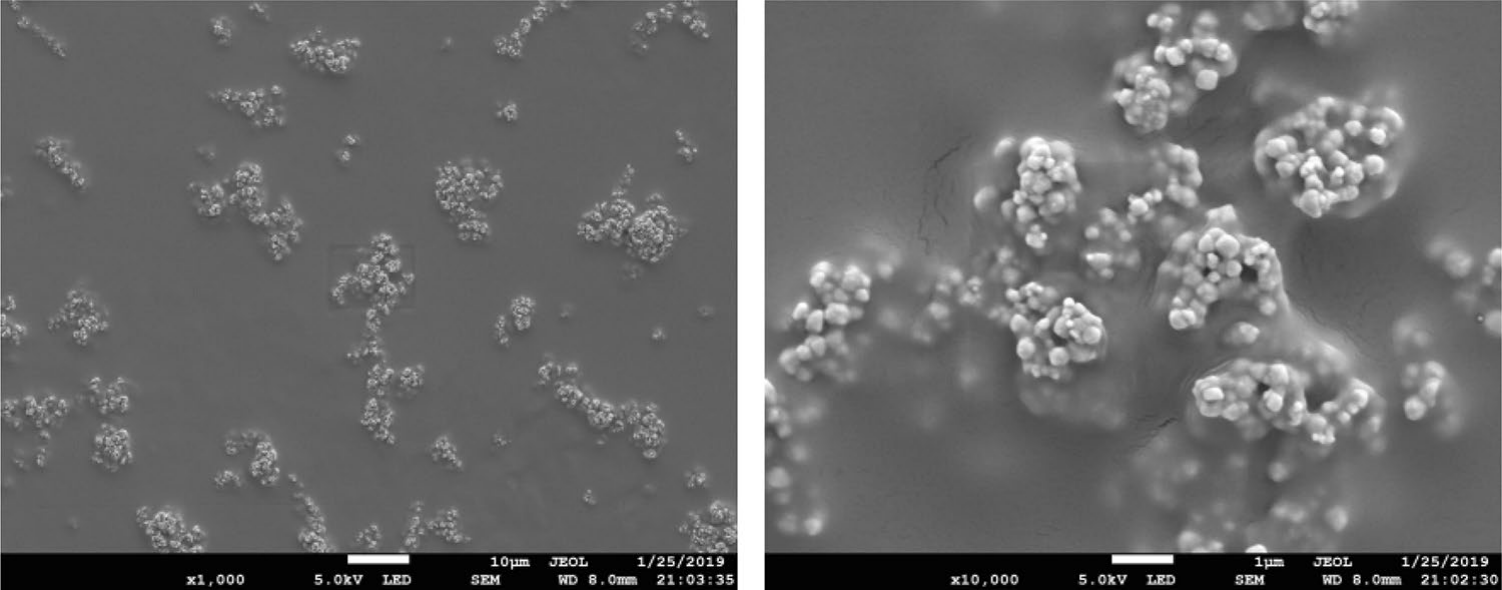
Scanning electron microscope of magnetic beads preset coating.

The scanning electron microscope (SEM) of magnetic beads preset coating is shown in Fig. 6. It can be seen that the magnetic beads were tightly wrapped by water-soluble materials and attached to the bottom of magnetic beads preset coating groove. In this experiment, polyvinyl alcohol (PVA) with good biocompatibility and no environmental pollution was selected as water-soluble material.

### Preset magnetic beads dissolution and capture

For the convenience of observation, quartz glass square hole capillary was used as the carrier for preset magnetic beads to observe the effect of dissolution and capture.

1. Materials and equipment: Quartz glass square hole capillary with inner diameter of 1 × 1 mm; Nd-Fe-B strong permanent magnet with external dimension of 20 × 10 × 3 mm; Video microscope, ZX-H1400, Shenzhen Zhongwei Kechuang Technology Co., Ltd.
2. Experimental methods: a wire with smooth surface, good centrality and diameter of 0.6 mm was placed in the quartz glass tube in advance. A certain concentration of PVA solution mixed evenly with magnetic beads was inhaled into a needle tube, then the PVA/magnetic beads mixture were injected into the quartz glass tube. After drying, the wire was carefully taken out from the quartz glass tube to form a preset magnetic beads quartz glass tube. The quartz glass tube was placed in the self-made bracket with one end connected with the plunger pump and the other end led into the beaker through the teflon tube. A permanent magnet was set under the quartz glass tube, and a video microscope was set in front of the quartz glass tube. After all the preparations were done, start the plunger pump to make the distilled water pass through the quartz glass tube at a certain flow rate and observe the dissolution effect.

**Figure 7:**
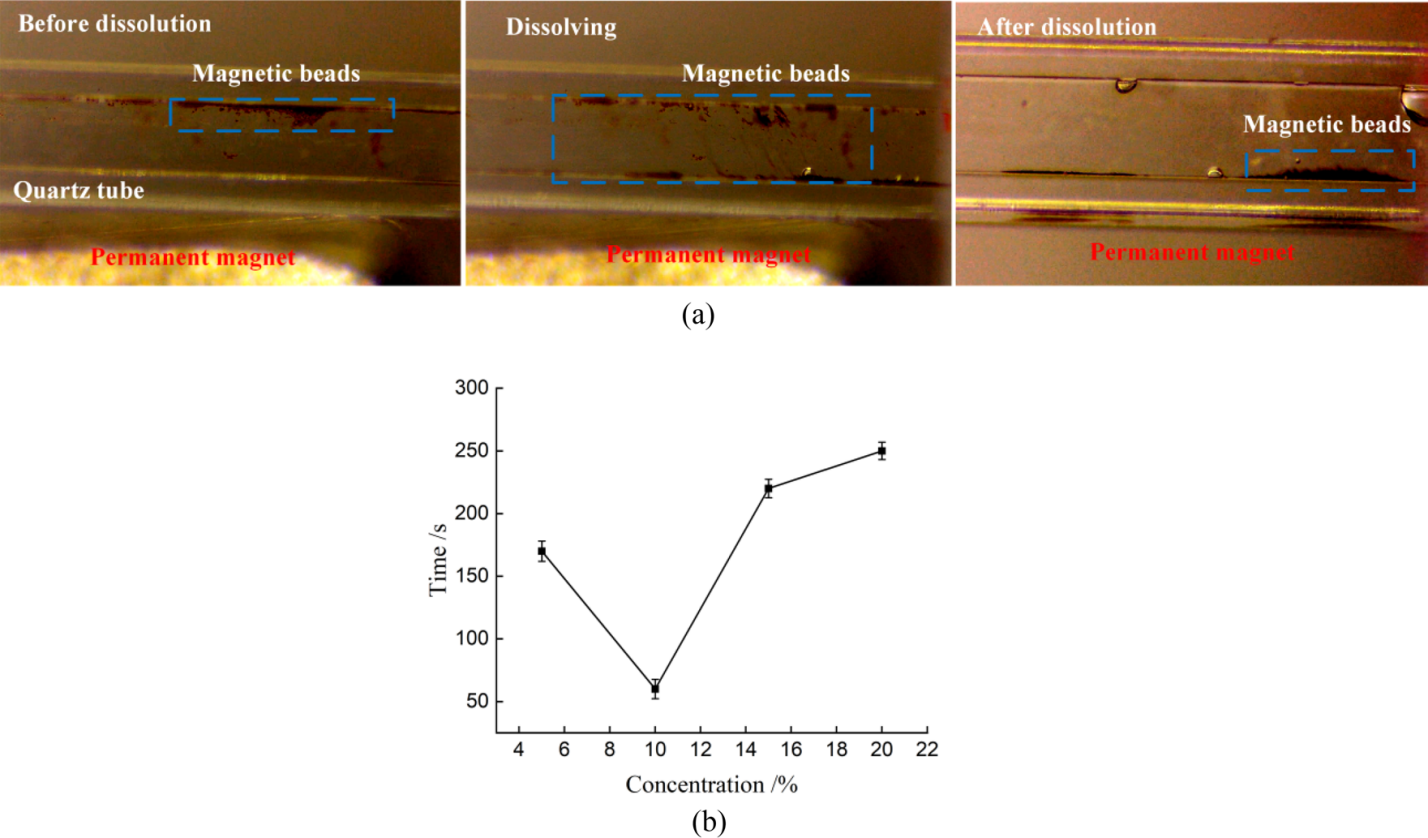
Dissolution effect. (**a**) Magnetic bead dissolution process. (**b**) Dissolution time of PVA with different concentrations.

The dissolving process of preset magnetic beads is shown in Fig. 7a. The dissolution rate of magnetic beads is related to the amount of preset magnetic beads, the solubility of PVA solution, the size of permanent magnet and the flow rate. The effect of different PVA solution concentrations on the complete release time of magnetic beads is shown in Fig. 7b, from which can be seen that with the increase of concentration, the PVA complete dissolution time first decreases and then increases. When the concentration is 10%, the complete dissolution time is the smallest. That is because, with the increase of concentration, the more PVA dosage is used, the smaller molecules are formed in solution to make fully dissolve, and shorten the dissolution time. When the concentration exceeds 10%, the concentration of solute is too high, which hinders the expansion of PVA small molecules, and prolongs the dissolution time. However, the experimental results showed that the preset magnetic beads can be completely dissolved and captured within 20 s by setting reasonable parameters.

### Function verification of preset magnetic beads

**Figure 8:**
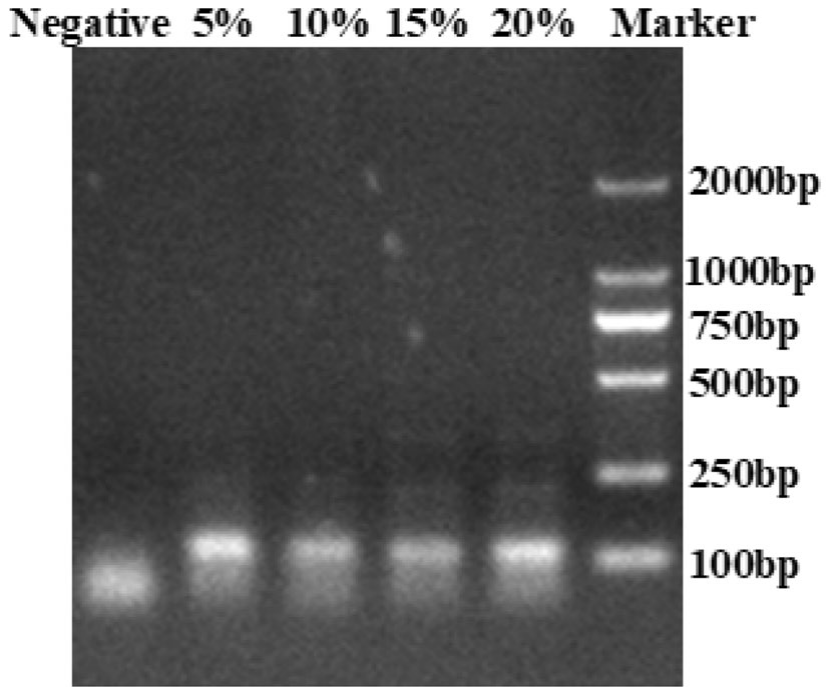
Electrophoretic detection of magnetic beads adsorption ability under different PVA concentration.

It should be verified whether there was change in the function of magnetic beads mixed with PVA solution or not. The magnetic beads were prefabricated with 5%, 10%, 15%, 20% PVA coating solution respectively, then dissolved and removed PVA to acquire preset magnetic beads. The preset magnetic beads adsorbed the same plasmid DNA containing HBV genome, then the extracted DNA was detected by PCR. After reaction, 5 μL reaction products were detected by the electrophoresis with 2% agarose gel to evaluate whether PVA affected the adsorption ability of magnetic beads. The electrophoretic detection of magnetic beads adsorption ability under different PVA concentration are shown in Fig. 8. The detect results showed that there was no significant difference in the position and brightness of the four bands of reaction products with different concentrations (5%, 10%, 15% and 20%). Through the relevant experiments, it can be confirmed that the detection effect of magnetic beads dissolved from the PVA solution is consistent with those of ordinary magnetic beads, indicating that the magnetic beads dissolved from the PVA solution do not affect their surface function.

## Analysis of preset magnetic beads motion characteristics

### Coupled field analysis

The dynamic characteristics of magnetic particles are mainly determined by two factors in microfluidic chips, the gradient magnetic field applied externally and the flow field in the microchannel. Therefore, the motion characteristics of magnetic beads in the microchannel mainly involve the theory of magnetostatics, hydrodynamics and Newton's second law.

#### Magnetic field

Magnetic field is the main way to control material transport in microfluidic chip. Compared with electric field, magnetic field has unique advantages: magnetic force is not affected by sample concentration, pH value and other parameters; No heat is generated by permanent magnet; Magnetic field can penetrate most of the materials such as glass and polymer^36^; magnetic field is mild and non-destructive to most living cells. In magnetic beads preset technology, compound magnetic field effect of different permanent magnets on the magnetic beads is mainly considered.

#### Flow field

When the magnetic beads enter the chip from outside, the capture efficiency of magnetic beads is closely related to the flow rate. When the flow rate is low, the capture efficiency is high, but the efficiency of biological analysis system is low. When the flow rate is high, the capture efficiency is low. Although the suitable flow rate and capture efficiency can be achieved with optimizing, using the magnetic beads preset technology can weaken the influence of flow rate, focus on the influence of static flow field on magnetic beads, and simplify the analysis model.

### Mathematical model of magnetic beads motion

The external forces on magnetic beads in the microchannel include magnetic force, fluid viscous force, interaction force between particles, gravity and buoyancy^37,38^. According to Newton's second law:

1 $$ m\frac{{d^{2} \tau }}{{dt^{2} }} = F_{m} + F_{d} + F_{g} + F_{f} $$

where *m* is the mass of particle, *τ* is the position vector diameter of particle, and *F*_*m*_, *F*_*d*_, *F*_*g*_, *F*_*f*_ are the magnetic force, viscous force, gravity and buoyancy. Since magnetic force and viscous force are much greater than gravity and buoyancy, the model is simplified as formula (2), ignoring gravity and buoyancy.

2 $$ m\frac{{d^{2} \tau }}{{dt^{2} }} = F_{m} + F_{d} $$

**Figure 9:**
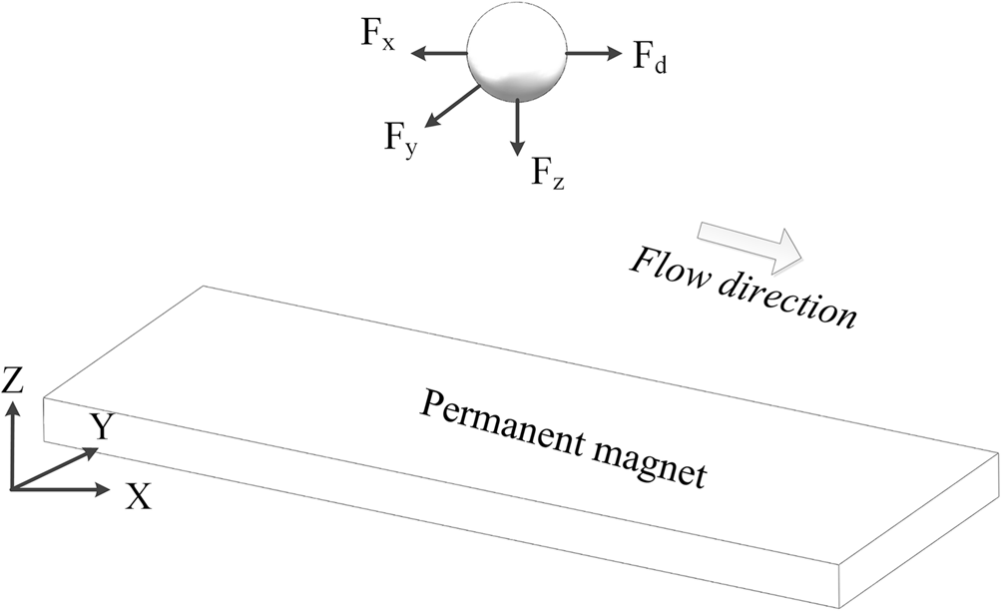
Forces of magnetic bead.

The forces on magnetic bead are shown in Fig. 9. The magnetic field force is decomposed into three components (*F*_*x*_, *F*_*y*_, *F*_*z*_).

#### Magnetic force *F*_*m*_

Magnetic force *F*_*m*_ can be expressed as:

3 $$ F_{m} = \mu_{0} MV_{p} \nabla H $$

where *μ*_*0*_ is the vacuum permeability, *M* is the field dependent magnetization of magnetic bead, *V*_*p*_ is the volume of magnetic bead, and *H* is the space magnetic field strength. When the particle is saturated magnetized, its field dependent magnetization is the saturation magnetization, that is, *M* = *M*_*s*_.

In general, the field dependent magnetization follows the formula:

4 $$ M = f(H)H $$

where *H* is the mode of magnetic field intensity, *H* = *|h|*, and *f(H)* satisfies the equation:

5 $$ f(H) = \left\{ {\begin{array}{*{20}l} {\frac{3\chi }{{3 + \chi }},} \hfill & {H > \frac{\chi + 3}{{3\chi }}M} \hfill \\ {\frac{{M_{s} }}{H} = \chi ,} \hfill & {H \ge \frac{\chi + 3}{{3\chi }}M} \hfill \\ \end{array} } \right. $$

where, $V_{p} = \frac{3}{4}\pi r^{3}$, $\chi$ is the magnetic susceptibility of the magnetic bead, and *R* is the radius of the magnetic bead.

Because $$H = \frac{B}{{\mu_{0} }}$$, so:

6 $$ F_{m} = \frac{1}{{\mu_{0} }}f(H)V_{p} B\nabla B{ = }\frac{1}{{\mu_{0} }}f(H)V_{p} \left( {B_{x} \frac{\partial B}{{\partial x}} + B_{y} \frac{\partial B}{{\partial y}} + B_{z} \frac{\partial B}{{\partial z}}} \right) $$

where *B* is the magnetic flux density.

The magnetic field force on magnetic bead depends not only on the magnetic induction intensity, but also on the magnetic field gradient. Magnetic bead tends to move towards the maximum magnetic field intensity in the non-uniform magnetic field. The magnetic force in different directions are as follows:

7 $$ \left\{ \begin{gathered} F_{{\text{x}}} = \frac{1}{{\mu_{0} }}f(H)V_{p} B\nabla B{ = }\frac{1}{{\mu_{0} }}f(H)V_{p} \left( {B_{x} \frac{{\partial B_{x} }}{\partial x} + B_{y} \frac{{\partial B_{x} }}{\partial y} + B_{z} \frac{{\partial B_{x} }}{\partial z}} \right) \hfill \\ F_{y} = \frac{1}{{\mu_{0} }}f(H)V_{p} B\nabla B{ = }\frac{1}{{\mu_{0} }}f(H)V_{p} \left( {B_{x} \frac{{\partial B_{y} }}{\partial x} + B_{y} \frac{{\partial B_{y} }}{\partial y} + B_{z} \frac{{\partial B_{y} }}{\partial z}} \right) \hfill \\ F_{{\text{z}}} = \frac{1}{{\mu_{0} }}f(H)V_{p} B\nabla B{ = }\frac{1}{{\mu_{0} }}f(H)V_{p} \left( {B_{x} \frac{{\partial B_{z} }}{\partial x} + B_{y} \frac{{\partial B_{z} }}{\partial y} + B_{z} \frac{{\partial B_{z} }}{\partial z}} \right) \hfill \\ \end{gathered} \right. $$

#### Viscous force *F*_*d*_

In microfluidic system with low Reynolds number, the viscous force acting on particles can be obtained by Stokes law:

8 $$ F_{d} = 6\pi \eta r(v_{f} - v_{p} )f_{D} $$

where, *η* is the dynamic viscosity of fluid, *r* is the radius of magnetic bead, *v*_*f*_ is the velocity of fluid, *v*_*p*_ is the velocity of magnetic bead, and *f*_*d*_ is the hydrodynamic resistance coefficient.

9 $$ v_{p} = \frac{d\tau }{{dt}} $$

When the wall effect is considered, the expression of hydrodynamic resistance coefficient is as follows:

10 $$ f_{D} = \left[ {1 - \frac{9}{16}\left( {\frac{r}{r + s}} \right) + \frac{1}{8}\left( {\frac{r}{r + s}} \right)^{3} - \frac{45}{{256}}\left( {\frac{r}{r + s}} \right)^{4} - \frac{1}{16}\left( {\frac{r}{r + s}} \right)^{5} } \right]^{ - 1} $$

where *s* is the distance between the magnetic bead and the wall of microchannel. When the magnetic bead is far away from the wall, *f*_*D*_ = *1*; When the magnetic bead is near the wall, *f*_*D*_ is slightly greater than 1.

#### Speed of magnetic bead

The time required for magnetic bead to reach a new equilibrium is extremely small. It can be considered that the motion of magnetic bead in the microchannel is always in a Quasi equilibrium state. The motion equation of the magnetic bead can be simplified as:

11 $$ F_{m} + F_{d} { = }0 $$

By introducing Eqs. (8) and (9), we get the following results:

12 $$ v_{p} = \frac{d\tau }{{dt}} = \frac{1}{{6\pi \eta rf_{D} }}F_{m} + v_{f} $$

The velocity components in each direction can be expressed as:

13 $$ \left\{ \begin{gathered} v_{px} = \frac{dx}{{dt}} = \frac{1}{{6\pi \eta rf_{D} }}F_{mx} + v_{fx} \hfill \\ v_{py} = \frac{dy}{{dt}} = \frac{1}{{6\pi \eta rf_{D} }}F_{my} + v_{fy} \hfill \\ v_{pz} = \frac{dz}{{dt}} = \frac{1}{{6\pi \eta rf_{D} }}F_{mz} + v_{fz} \hfill \\ \end{gathered} \right. $$

When the magnetic beads with a certain velocity flows go through the microchannel, the magnetic beads will be shifted and separated from the fluid by magnetic force. Considering that the width to height ratio of the microchannel is large, the separation process can be simplified as a 2D model.

### Motion analysis of preset magnetic bead capture

**Figure 10:**
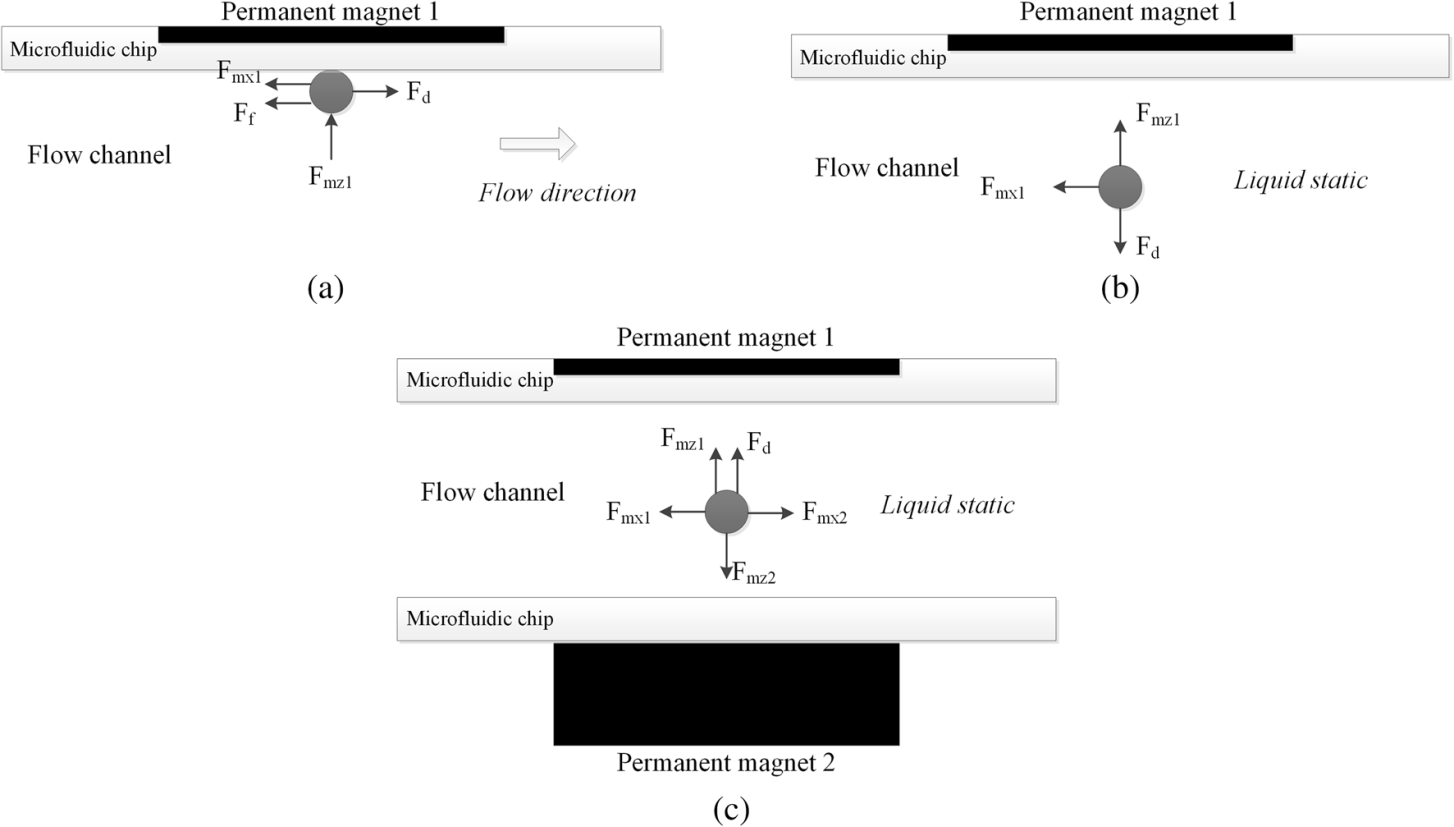
Force analysis of preset magnetic beads. (**a**) Preset magnetic bead capture. (**b**) Upward mixing motion. (**c**) Downward mixing motion.

The capture motion includes magnetic beads captured by permanent magnet 1 (preset permanent magnet) after magnetic beads released and fixed on the upper surface of the channel during solution washing. The force analysis of magnetic bead capture is shown in Fig. 10a, where *F*_*f*_ is the friction force between magnetic bead and the channel upper surface. When the magnetic bead is stable in the capture position, *F*_*mx1*_ = *0* and *v*_*P*_ = *0*. The condition for the successful capture of magnetic beads under the velocity *v*_*f*_ is as follows:

14 $$ F_{f} { = }F_{{\text{d}}} $$

In the critical state:

15 $$ F_{f} = fF_{{{\text{mz1}}}} $$

where *f* is the friction coefficient between the magnetic bead and the flow channel. Introduce Eqs. (8) and (15) into Eq. (14), and get:

16 $$ F_{mz1} { = }\frac{{6\pi \eta rf_{D} }}{f}v_{f} $$

Let $\frac{{6\pi \eta rf_{D} }}{f} = [{\text{a}}]$, When the microfluidic system is determined, [a] is a certain value, then:

17 $$ F_{mz1} = [a]v_{f} $$

Therefore, only when the z-direction magnetic force produced by permanent magnet 1 is greater than [a] times of the liquid flow rate, the magnetic beads can be effectively captured.

### Motion analysis of preset magnetic bead mixing

The mixing motion between magnetic beads and solution can be divided into upward mixing motion and downward mixing motion, which are driven by different permanent magnets. Because of using preset magnetic beads technology, *v*_*fz*_ is considered as *0*.

#### Upward mixing motion

The upward mixing motion is achieved by permanent magnet 1. At this time, the permanent magnet 2 falls far enough, so the influence of its magnetic field on magnetic bead can be ignored. The force analysis of upward mixing motion of magnetic bead is shown in Fig. 10b. The achievement of upward mixing motion should satisfy *F*_*mz1*_ > *F*_*d*_, considering the quasi equilibrium state of magnetic bead motion:

18 $$ v_{pz1} = \frac{dz}{{dt}} = \frac{1}{{6\pi \eta rf_{D} }}F_{mz1} $$

The microchannel height is *Q*, and integrate both sides:

19 $$ t_{1} = \frac{{6\pi \eta rf_{D} Q}}{{F_{mz1} }} $$

where *t*_*1*_ is the time required for upward mixing motion.

#### Downward mixing motion

The downward mixing motion is achieved by permanent magnet 2. The force analysis of downward mixing motion of magnetic bead under the influence of the magnetic field of permanent magnet 1 is shown in Fig. 10c. The achievement of downward mixing motion should satisfy *f*_*mz2*_ > *f*_*mz1*_ + *F*_*d*_, considering the quasi equilibrium state of magnetic bead motion:

20 $$ \left\{ \begin{gathered} v_{pz1} = \frac{1}{{6\pi \eta rf_{D} }}F_{mz1} \hfill \\ v_{pz2} = \frac{1}{{6\pi \eta rf_{D} }}F_{mz2} \hfill \\ \end{gathered} \right. $$

The downward velocity of magnetic bead is:

21 $$ v_{pz} = \frac{dz}{{dt}}{ = }v_{pz2} - v_{pz1} = \frac{1}{{6\pi \eta rf_{D} }}(F_{mz2} - F_{mz1} ) $$

Integrate both sides:

22 $$ t_{2} = \frac{{6\pi \eta rf_{D} Q}}{{F_{mz2} { - }F_{mz1} }} $$

where *t*_*2*_ is the time required for downward mixing motion.

The mixing efficiency of magnetic beads is determined by *t*_*1*_ and *t*_*2*_. After the magnetic beads are constant, the mixing efficiency depends on the following factors: (1) the smaller fluid dynamic viscosity, the higher mixing efficiency; (2) the greater magnetic field intensity of preset permanent magnet, the shorter upward mixing motion time, but the downward mixing motion needs to be considered comprehensively; (3) the greater magnetic field intensity difference, the shorter downward mixing motion time; (4) the smaller channel height, the higher mixing efficiency. After the type of magnetic beads, the size and quantity of flow channel and the type of different reaction solutions are determined, the specification of permanent magnet should be reasonably selected to ensure the magnetic beads motion effect, simplify the device structure and reduce the structure size.

Through the derivation of the upward mixing movement time and downward mixing movement time of preset magnetic beads, the time required for a single full mixing movement can be clearly obtained, and the total time required for the mixing movement can be obtained according to the number of up-and-down movements through related DNA extraction and elution effect experiment, which provides a basis for the determination of relevant parameters of the control magnetic field.

### Numerical simulation for magnetic field

**Figure 11:**
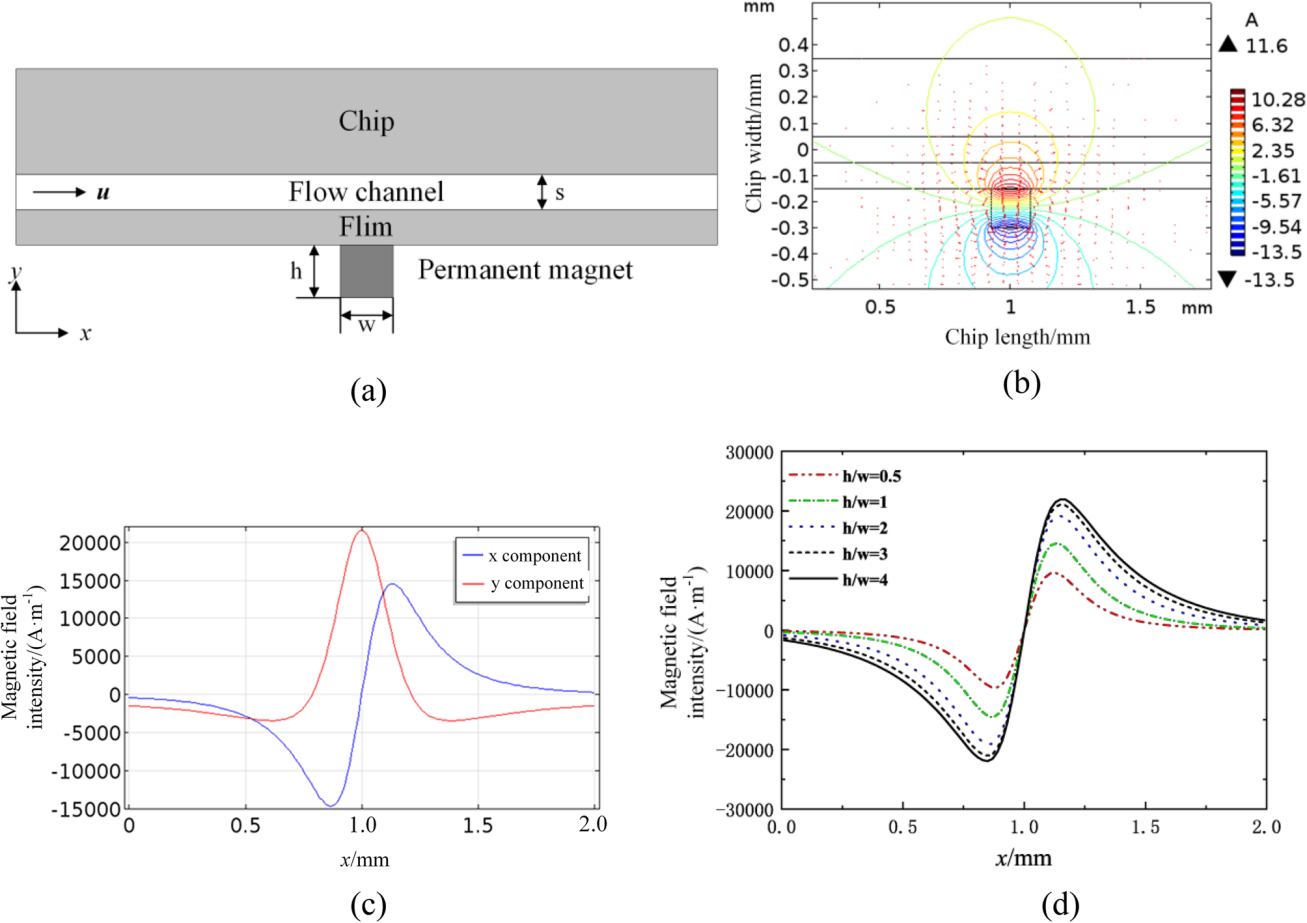
Numerical simulation for magnetic field. (**a**) 2D analysis model. (**b**) Spatial distribution of magnetic field. (**c**) Magnetic field intensity component in microchannel centerline. (**d**) Magnetic field *x* components of microchannel centerline with different aspect ratio.

In the magnetic beads preset technology, the most important is how to select the appropriate permanent magnet to ensure the capture efficiency and mixing efficiency. We have developed a method to simulate the magnetic field by using COMSOL and verify the variation characteristics of magnetic field by changing the aspect ratio of permanent magnet in the microfluidic channel. As shown in Fig. 11a, the 2D model was composed of permanent magnet, microchannel, chip and film. The related dimensions are: the length of microchannel 2 mm, the width of microchannel 0.1 mm, the permanent magnet initial dimension of width *w* = 0.15 mm and height *h* = 0.15 mm. The material of microfluidic channel was set as air, which could truly simulate the impact of the environment. The boundary condition of the air was set as magnetic insulation, and the grid was divided according to the standard. The relevant parameters are: relative permeability of permanent magnet 1, vacuum permeability 4*π* × 10^−7^ H A^−2^, permanent magnet x axial remanence 0, permanent magnet y axial remanence ± 0.4 T.

The spatial distribution of magnetic field is shown in Fig. 11b. The densest magnetic flux density region is concentrated in the interior of permanent magnet and gradually decreases upward along the radial direction of the channel. The gradient magnetic field in the microchannel above the permanent magnet is larger and the effect of the magnetic field extending to both sides is reduced. Therefore, the magnetic flux density near the magnet direction in the microchannel is larger and the magnetic field is stronger.

The magnetic field intensity component generated by permanent magnet in microchannel centerline is shown in Fig. 11c. The magnetic field gradient in x-direction is the largest near *x* = 1 mm, and the magnetic field intensity in y-direction reaches the maximum. Since the magnetic field force is directly proportional to the magnetic field gradient, it can be inferred that the magnetic force in x-direction will have the maximum near *x* = 1 mm. Therefore, the magnetic beads will tend to move in the central region of the permanent magnet.

Magnetic field *x* components of microchannel centerline with different aspect ratio are shown in Fig. 11d. When the width *w* of the permanent magnet remains constant, the aspect ratio has no effect on the curve shape of the magnetic field intensity, which is a sinusoidal curve. The maximum value of x-direction magnetic field intensity and gradient magnetic field increase with the increase of magnet height *h*, but when the height of permanent magnet increases to a certain value, the increase of magnetic field intensity in the channel decreases gradually until it keeps constant. Therefore, the aspect ratio of permanent magnet can be increased appropriately to obtain greater magnetic field force and improve the magnetic beads capture and mixing efficiency.

## Conclusion

A magnetic beads preset technology was provided, and the feasibility of its application in microfluidic chip was verified. Based on the analysis of magnetic beads control motion, the motion model of preset magnetic beads was established, including capture motion and mixing motion. The mixing motion was decomposed into upward mixing motion and downward mixing motion, and the relationship between capture condition, mixing time and coupling field was derived, which can provide a calculation and design basis for simulation and control of preset magnetic beads. However, in the derivation of mixing time, the influence of movable permanent magnet was completely ignored in the upward mixing motion. And the time of downward mixing motion was calculated based on the movable permanent magnet moving to the closest position to the chip, but the equilibrium position should be located in a certain position during the ascending motion of movable permanent magnet. The actual mixing time should be shorter than the calculation time, that is, the velocity of movable permanent magnet need to be considered for a more accurate magnetic beads motion control time. In addition, our study ignored the influence of magnetic force in the direction of flow channel, which can be considered in the follow-up study to deeply analyze the distribution trajectory of magnetic beads along the flow channel.

## References

[1] Hohnholt MC (2011). Treatrent with iron oxide nanoparticles induces ferritin synthesis but not oxidative stress in oligodendroglial cell. Acta Biomater..

[2] Lim CT, Zhang Y (2007). Bead-based microfluidic immunoassays: The next generation. Biosens. Bioelectron..

[3] Safarík I, Safaríková M (1999). Use of magnetic techniques for the isolation of cells. J. Chromatogr. B Biomed. Sci. Appl..

[4] Safarik I, Safarikova M (2004). Magnetic techniques for the isolation and purification of proteins and peptides. Biomagn. Res. Technol..

[5] Esmaeili E (2017). Hybrid magnetic-DNA directed immobilisation approach for efficient protein capture and detection on microfluidic platforms. Sci. Rep..

[6] Bao G (2013). Multifunctional nanoparticles for drug delivery and molecular imaging. Annu. Rev. Biomed. Eng..

[7] Singh A, Sahoo SK (2014). Magnetic nanoparticles: A novel platform for cancer theranostics. Drug Discov. Today..

[8] Song X (2015). Preparation and characterization of biofunctionalized chitosan/Fe_3_O_4_ magnetic nanoparticles for application in liver magnetic resonance imaging. J. Magn. Magn. Mater..

[9] Hejazian M, Nguyen N-T (2015). Negative magnetophoresis in diluted ferrofluid flow. Lab Chip..

[10] Zhu T (2012). Continuous-flow ferrohydrodynamic sorting of particles and cells in microfluidic devices. Microfluid. Nanofluid..

[11] Xi Z (2015). Selection of HBsAg-specifc DNA aptamers based on carboxylated magnetic nanoparticles and their application in the rapid and simple detection of hepatitis B virus infection. ACS Appl. Mater. Interfaces..

[12] Alnaimat F (2018). Microfluidics based magnetophoresis: A review. Chem. Rec..

[13] Reenen AV (2014). Integrated lab-on-chip biosensing systems based on magnetic particle actuation—A comprehensive review. Lab Chip.

[14] Zhou R, Wang C (2016). Microfluidic separation of magnetic particles with soft magnetic microstructures. Microfluid Nanofluid..

[15] Zhu Y (2020). Magnetic beads separation characteristics of a microfluidic bioseparation chip based on magnetophoresis with lattice-distributed soft magnets. J. Magn. Magn. Mater..

[16] Serra M (2019). Integrated droplet microfluidic device for magnetic particles handling: Application to DNA size selection in NGS libraries preparation. Sensors Actuators B Chem..

[17] Munaz A (2018). Magnetophoretic separation of diamagnetic particles through parallel ferrofluid streams. Sensors Actuators B Chem..

[18] Zhang P (2019). Ultrasensitive detection of circulating exosomes with a 3D-nanopatterned microfluidic chip. Nat. Biomed. Eng..

[19] Zheng LY (2019). Optical biosensor for rapid detection of salmonella typhimurium based on porous gold@platinum nanocatalysts and a 3D fluidic chip. ACS Sensors..

[20] Myklatun A (2017). Microfluidic sorting of intrinsically magnetic cells under visual control. Sci. Rep..

[21] Cao Q (2017). Dynamic motion analysis of magnetic particles in microfluidic systems under an external gradient magnetic field. Microfluid. Nanofluid..

[22] Pourmehran O (2016). Magnetic drug targeting through a realistic model of human tracheobronchial airways using computational fluid and particle dynamics. Biomech. Model. Mechanobiol..

[23] Zeng J (2013). Magnetic separation of particles and cells in ferrofluid flow through a straight microchannel using two offset magnets. J. Magn. Magn. Mater..

[24] Tarn MD (2009). The importance of particle type selection and temperature control for on-chip free-flow magnetophoresis. J. Magnet. Magnet. Mater..

[25] Ghazali FAM (2020). MEMS actuators for biomedical applications: A review. J. Micromech. Microeng..

[26] Zhou R (2016). Fabrication and integration of microscale permanent magnets for particle separation in microfluidics. Microfluid. Nanofluid..

[27] Yang C, Li G (2017). A novel magnet-actuated droplet manipulation platform using a floating ferrofluid film. Sci. Rep..

[28] Guckenberger DJ (2016). Magnetic system for automated manipulation of paramagnetic particles. Analyt. Chem..

[29] Pamma N (2006). Magnetism and microfluidics. Lab Chip.

[30] Antfolk M, Laurell T (2017). Continuous flow microfluidic separation and processing of rare cells and bioparticles found in blood—A review. Anal. Chim. Acta.

[31] Jayamohan H (2017). Advances in microfluidics and lab-on-a-chip technologies. Mol. Diagnostics..

[32] Schuler F (2015). Digital droplet PCR on disk. Lab Chip.

[33] Sun Y (2015). A lab-on-a-chip system with integrated sample preparation and loop-mediated isothermal amplification for rapid and quantitative detection of *Salmonella* spp. in food samples. Lab Chip..

[34] Cao W (2015). Automated microfluidic platform for serial polymerase chain reaction and high-resolution melting analysis. J. Lab. Autom..

[35] Shi X (2015). Parallel RNA extraction using magnetic beads and a droplet array. Lab Chip..

[36] Suwa M, Watarai H (2011). Magnetoanalysis of micro/nanoparticles: A review. Anal. Chim. Acta.

[37] Banerjee U (2012). Aggregation dynamics of particles in a microchannel due to an applied magnetic field. Microfluid. Nanofluid..

[38] Mdali MA (2016). Active bioparticle manipulation in microfluidic systems. RSC Adv..

